# Neglected Gallstone Disease Presented As Gallstone Ileus: A Rare Cause of Intestinal Obstruction

**DOI:** 10.7759/cureus.18205

**Published:** 2021-09-23

**Authors:** Navin Kumar, Rohik Anjum, Rishit Mani, Bibek Karki

**Affiliations:** 1 General Surgery, All India Institute of Medical Sciences, Rishikesh, IND

**Keywords:** gall stone complications, gall stone disease (gsd), cholecystoduodenal fistula, intestinal obstruction, gall stone ileus

## Abstract

Gallstone ileus is a rare complication of cholelithiasis seen in patients with a long history of cholelithiasis. It occurs more in the older age group and in the female gender. These patients have poor general condition and therefore selection of appropriate treatment is difficult. The clinician has to make a decision between immediate one-stage or two-stage closure of the cholecysto-intestinal fistula or waiting for natural closure. We have discussed the management of a rare cause of small bowel obstruction due to complication of untreated cholelithiasis.

## Introduction

Gallstone ileus is a rare complication of cholelithiasis [1]. Gall stone ileus is seen in 0.5% of patients with a long history of cholelithiasis, which occurs more in the older age group (> 60years) and in the female gender (3.5:1) [2]. In these patients, gallstones are migrated by internal biliary fistula. They are the most common route of elimination from the gallbladder to the duodenum (75-83%) but they may also be eliminated through the colon or stomach. The most common site of impaction of gallstone is the ilium (50-60%) followed by jejunum (16.1-26.9%), duodenum (3.5-14.6%), and colon (3-4.1%) [3]. These patients have poor general conditions and therefore selection of appropriate treatment is difficult. The clinician has to make a decision between immediate one-stage or two-stage closure of the cholecysto-intestinal fistula or waiting for natural closure. 

## Case presentation

**Figure 1:**
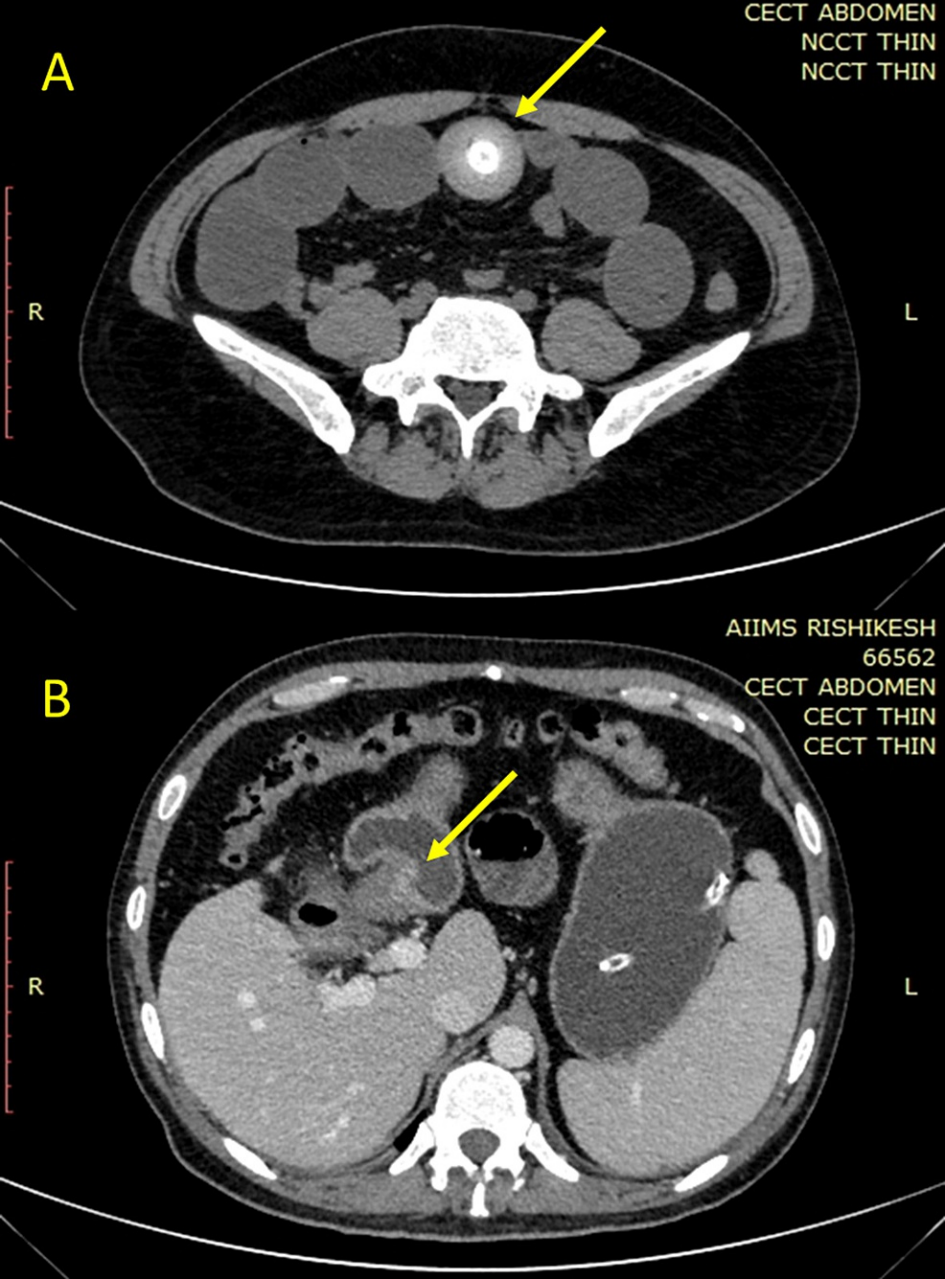
CT scan of abdomen showing (A) dilated small bowel loops with lamellated hyperdense calculus ~17x15mm in lumen of distal jejunal loops at the transition point with collapsed distal bowel, and (B) thickened gallbladder wall with fistulous communication between the gallbladder and the duodenum

A 27-year-old man from North India*, *a prevalent zone for cholelithiasis, presented to surgery emergency with recurrent episodes of bilious vomiting and right upper abdominal pain of two days, followed by progressive abdominal distension with non-passage of flatus and feces for one day. He was diagnosed with symptomatic gallstone disease after an episode of biliary colic nine months back with normal laboratory values of liver enzymes (total bilirubin 0.8mg/dl, aspartate aminotransferase (AST) 30 IU/L, alanine aminotransferase (ALT) 32 IU/L, alkaline phosphatase (ALP) 115 IU/L, and gamma-glutamyl transferase (GGT) of 25 IU/L) for which he used to take analgesics on and off. There was no history of any other co-morbidities or past surgeries. His initial observation showed a heart rate of 110 beats/min and blood pressure of 126/70mmHg with abdominal distension and tenderness in the epigastrium and right hypochondrium. Blood investigations showed raised total leukocyte counts (20,000/cumm) and deranged liver function (bilirubin 2.1mg/dl, direct bilirubin 1.5mg/dl, alkaline phosphatase 321 IU/L, GGT of 79 IU/L, and normal ALT 42 IU/L and AST 32 IU/L). Abdominal radiography revealed features of small bowel obstruction. Contrast-enhanced computed tomography (CECT) scan of the abdomen with diluted mannitol as oral contrast was done, which showed dilated small bowel loops (maximum diameter 3.8cm) with lamellated hyperdense calculus ~17x15mm in the lumen of distal jejunal loops at the transition point with collapsed distal bowel (Figure 1 A). There was associated pneumobilia in intra- and extra-hepatic biliary radicals with fistulous tract between the gallbladder and the duodenum (Figure 1 B).

**Figure 2:**
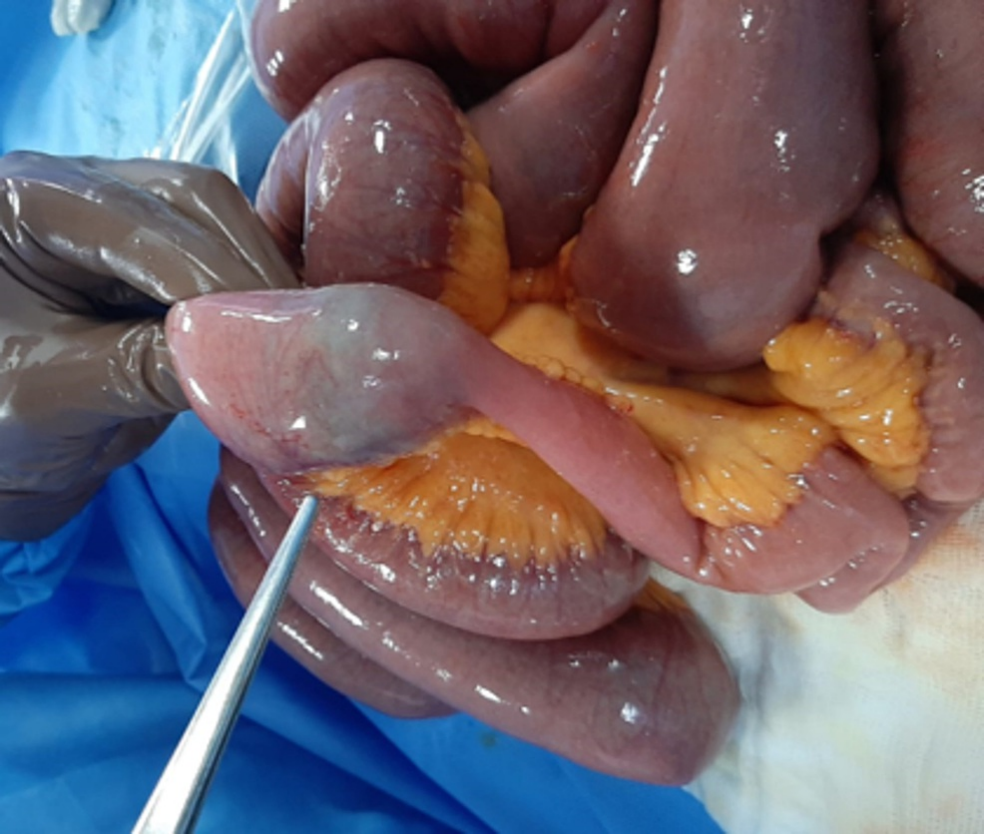
Intraoperative image showing the transition point between dilated proximal jejunum and distal collapsed bowel. The calculus can be seen through the wall of the bowel

The provisional diagnosis of gallstone ileus was made. After adequate resuscitation with intravenous fluid and broad-spectrum antibiotic, an emergency exploratory laparotomy was done through a midline incision. Intraoperatively small bowel loops were dilated with an impacted stone of ~3x3cm in the distal jejunum (Figure 2) and inflamed gallbladder with adhered first part of duodenum with fistulous communication. 

**Figure 3:**
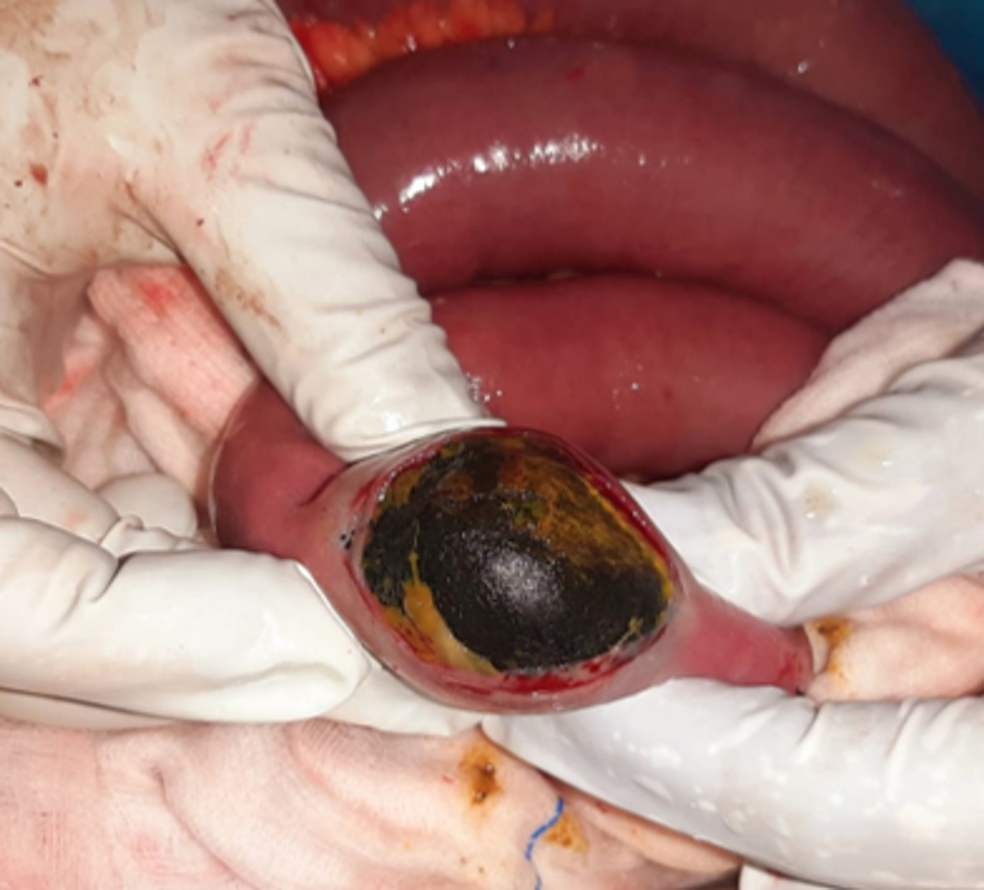
Intraoperative image of longitudinal enterotomy with the calculus

**Figure 4:**
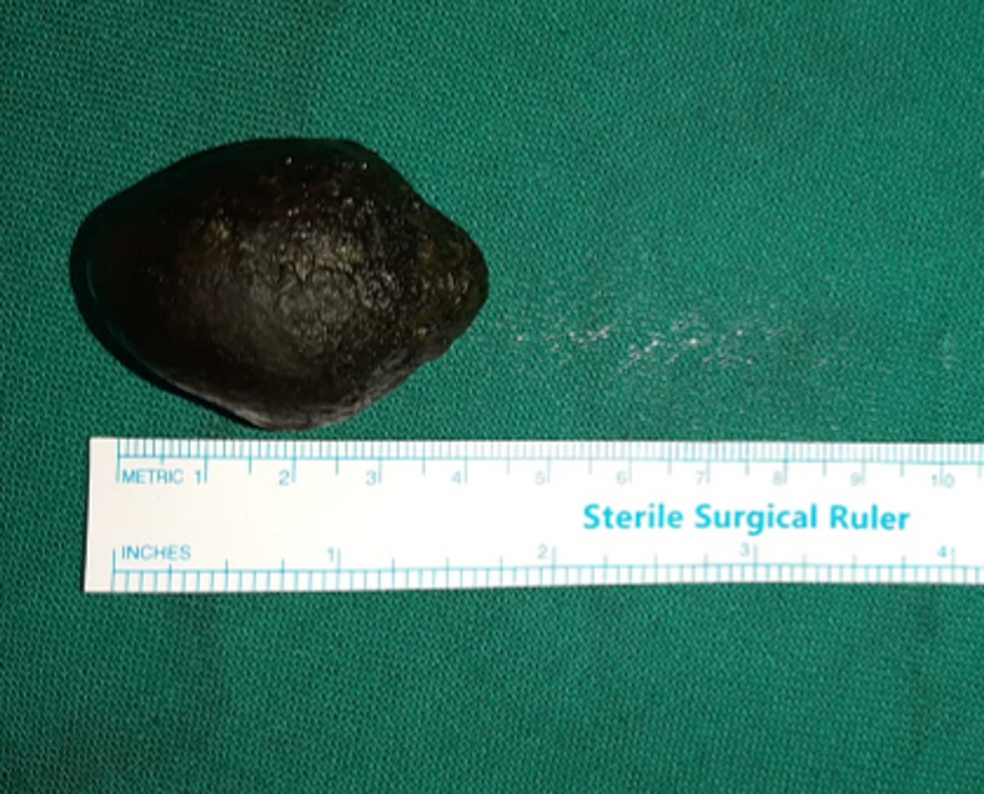
intraoperative image of calculus (4cmx2.5cm)

A longitudinal enterotomy was made on the bowel over the calculus, and the stone was extracted out (Figure 3 and Figure 4).

**Figure 5:**
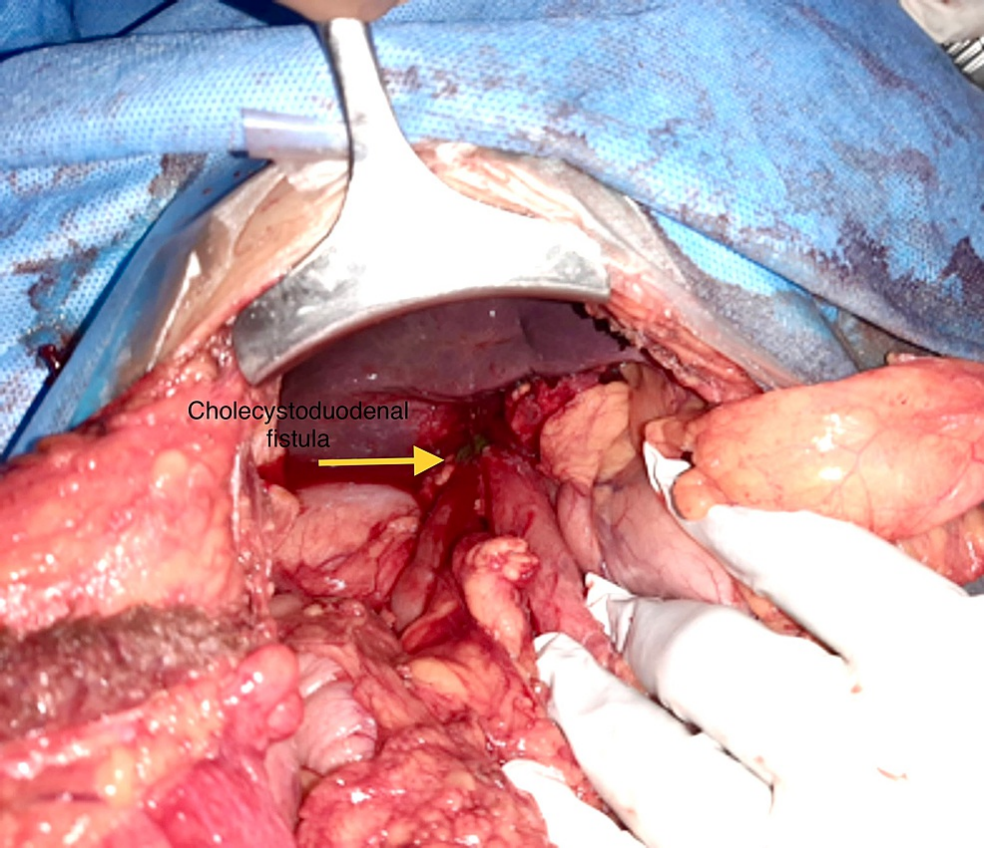
Intraoperative image showing inflamed gallbladder with adhered first part of duodenum with fistulous communication (cholecystoduodenal fistula)

The enterotomy was closed in the horizontal direction (Heineke-Mikulicz procedure), which was followed by cholecystectomy with excision of the fistulous tract (Figure 5). The duodenal defect was repaired with interrupted 2-0 polydioxanone sutures (PDS). The post-operative period was uneventful. Histopathological examination of gallbladder specimen was suggestive of chronic calculous cholecystitis.

## Discussion

Acute intestinal obstruction is one of the most common abdominal surgical emergencies seen in practice. Gallstone ileus as the cause of intestinal obstruction is an uncommon entity, seen in only 0.3-0.5% of patients who suffer from cholelithiasis, which occurs more in the older age group (> 60years) and in the female gender (3.5:1 ratio). In North India, cholelithiasis is prevalent and occurs at a younger age (as noted in our case). Other risk factors include a long history of cholelithiasis, repeated episodes of acute cholecystitis, and gallstone with size >2cm [2]. Dr. Erasmus Bartholin, a Danish physician, was the first person to describe this entity in 1654 [4]. The term gallstone ileus is a misnomer, as this condition occurs to small mechanical obstruction of the gut and is not a true ileus. In patients with a long-standing history of cholelithiasis with recurrent attacks of inflammation of the gall bladder, there is more adhesion between the gallbladder and the adjacent GI tract, which causes pressure necrosis and the formation of fistulous communication between these two structures. The most common GI tract site involved in the fistula is the duodenum due to the proximity between the two structures. However, there are reported cases of fistula formation between the transverse colon, stomach, jejunum, and ileum.

Once the fistula formation occurs, the gallstone migrates into the bowel and migrates down intraluminal with the peristalsis. Usually, a stone <2cm passes through the GI tract without any complications. However, larger stones tend to get impacted, with the most common site of impaction being the terminal ileum. The smallest diameter of the gut in the entire GI tract with reduced peristalsis and stasis due to the ileocaecal valve may cause impaction in the terminal ileum. In our case, the site of impaction was in the distal jejunum, probably owing to the larger size of the calculus [5]. Clinical features of this condition can be non-specific, including crampy abdominal pain, nausea, vomiting, and abdominal distension. The symptoms may be relieved intermittently when the stone 'tumbles' down the bowel. The degree of symptoms is directly proportional to the size of the stone and the luminal diameter of the bowel. Smaller stones (<2cm) may pass out of the rectum defecation without being noticed by the patient [6].

The diagnosis of this condition needs a high degree of clinical suspicion. Laboratory parameters can be non-specific, which includes leucocytosis, electrolyte imbalance, and elevated transaminase [3]. A plain radiograph may show features of intestinal obstruction with dilated bowel loops with multiple air-fluid levels. Rigler triad is a classically described sign to diagnose gallstone ileus, seen in 50% of cases. The appearance of pneumobilia, small bowel obstruction features, and gallstone appearance, usually in the right iliac fossa [7]. The gold standard imaging modality for the diagnosis of gallstone ileus is an abdominal CT scan. The findings consistent with the diagnosis of gallstone ileus include pneumobilia (30-60% cases), gallbladder thickening/ fistula formation between the gallbladder and adjacent GI tract, intestinal obstruction, and obstructing gallstones. A hepatobiliary iminodiacetic acid (HIDA) scan and magnetic resonance cholangiopancreatography (MRCP) are other imaging modalities that can be used [8].

The surgical treatment options for gall stone ileus are both one-stage procedure (cholecystectomy with fistula closure and enterotomy and stone extraction) and two-stage procedure (enterotomy and stone extraction followed by cholecystectomy and fistula closure at a later stage). However, two-stage procedure has a risk of cholecystitis, recurrence, and gallbladder malignancy due to persisting cholecysto-intestinal fistula. The single-stage procedure is recommended in low-risk patients with adequate nutrition and hemodynamic stability. For high-risk patients, a two-stage approach is recommended [9]. We have done a single-stage procedure because of the low-risk patient with good general condition.

## Conclusions

Gallstone ileus should be considered as a cause of bowel obstruction in a jaundiced patient with cholelithiasis. Abdominal CECT scan is diagnostic of gallstone ileus. In these cases biliary surgery can be carried out simultaneously with the intestinal obstruction (one-stage surgery) in low-risk cases, or performed later (two-stage surgery), or not at all in high-risk patients.

## References

[1] Halabi WJ, Kang CY, Ketana N (2014). Surgery for gallstone ileus: a nationwide comparison of trends and outcomes. Ann Surg.

[2] Inukai K, Uehara S, Miyai H (2018). Sigmoid gallstone ileus: a case report and literature review in Japan. Int J Surg Case Rep.

[3] Clavien PA, Richon J, Burgan S, Rohner A (1990). Gallstone ileus. Br J Surg.

[4] DE SL (1955). Gallstone ileus: a report of 12 cases. Ann Surg.

[5] Roade Tato L, Ventura Cots M, Riveiro-Barciela M (2018). Gallstone ileus secondary to a cholecystocolonic fistula [Article in English, Spanish]. Gastroenterol Hepatol.

[6] Masannat Y, Masannat Y, Shatnawei A (2006). Gallstone ileus: a review. Mt Sinai J Med.

[7] Gaduputi V, Tariq H, Rahnemai-Azar AA, Dev A, Farkas DT (2015). Gallstone ileus with multiple stones: where Rigler triad meets Bouveret's syndrome. World J Gastrointest Surg.

[8] Lassandro F, Romano S, Ragozzino A (2005). Role of helical CT in diagnosis of gallstone ileus and related conditions. AJR Am J Roentgenol.

[9] Reisner RM, Cohen JR (1994). Gallstone ileus: a review of 1001 reported cases. Am Surg.

